# Environmental bacterial load during surgical and ultrasound procedures in a Swedish small animal hospital

**DOI:** 10.1186/s13028-024-00768-4

**Published:** 2024-09-02

**Authors:** Todd Alsing-Johansson, Karin Bergström, Susanna Sternberg-Lewerin, Anna Bergh, Emma Östlund, Johanna Penell

**Affiliations:** 1 Department of Clinical Sciences, Faculty of Veterinary Medicine and Animal Science, Swedish University of Agricultural Sciences, 750 07 Uppsala, Sweden; 2 Department of Animal Health and Antimicrobial Strategies, Swedish Veterinary Agency, 751 89 Uppsala, Sweden; 3 Department of Animal Biosciences, Faculty of Veterinary Medicine and Animal Science, Swedish University of Agricultural Sciences, 750 07 Uppsala, Sweden; 4 Department of Microbiology, Swedish Veterinary Agency, 751 89 Uppsala, Sweden

**Keywords:** Antibacterial resistance, Antimicrobial resistance, Biosecurity, Contamination, Healthcare-associated infection, Hygiene, Infection prevention and control, Veterinary clinic

## Abstract

**Background:**

Environmental bacteria in animal healthcare facilities may constitute a risk for healthcare-associated infections (HAI). Knowledge of the bacterial microflora composition and factors influencing the environmental bacterial load can support tailored interventions to lower the risk for HAI. The aims of this study were to: (1) quantify and identify environmental bacteria in one operating room (OR) and one ultrasound room (UR) in a small animal hospital, (2) compare the bacterial load to threshold values suggested for use in human healthcare facilities, (3) characterise the genetic relationship between selected bacterial species to assess clonal dissemination, and (4) investigate factors associated with bacterial load during surgery.

Settle plates were used for passive air sampling and dip slides for surface sampling. Bacteria were identified by Matrix Assisted Laser Desorption—Time Of Flight. Antimicrobial susceptibility was determined by broth microdilution. Single nucleotide polymorphism-analysis was performed to identify genetically related isolates. Linear regression was performed to analyse associations between observed explanatory factors and bacterial load.

**Results:**

The bacterial load on settle plates and dip slides were low both in the OR and the UR, most of the samples were below threshold values suggested for use in human healthcare facilities. All settle plates sampled during surgery were below the threshold values suggested for use in human clean surgical procedures.

S*taphylococcus* spp. and *Micrococcus* spp. were the dominating species. There was no indication of clonal relationship among the sequenced isolates. Bacteria carrying genes conveying resistance to disinfectants were revealed.

Air change and compliance with hygiene routines were sufficient in the OR. No other factors possibly associated with the bacterial load were identified.

**Conclusions:**

This study presents a generally low bacterial load in the studied OR and UR, indicating a low risk of transmission of infectious agents from the clinical environment. The results show that it is possible to achieve bacterial loads below threshold values suggested for use in human healthcare facilities in ORs in small animal hospitals and thus posing a reduced risk of HAI. Bacteria carrying genes conveying resistance to disinfectants indicates that resistant bacteria can persist in the clinical environment, with increased risk for HAI.

**Supplementary Information:**

The online version contains supplementary material available at 10.1186/s13028-024-00768-4.

## Background

Recent studies suggest that environmental contamination, including pathogenic microorganisms on surfaces in direct contact with or near the patient, present a risk for healthcare-associated infections (HAI) in human healthcare facilities [1, 2] and presumably so also in animal healthcare facilities. Reported consequences of HAI in animal healthcare include prolonged hospital stays, as well as increased healthcare costs, morbidity and mortality [3]. Outbreaks of resistant bacteria such as carbapenemase-producing *Escherichia coli* [4]*,* clonal spread of a chlorhexidine-resistant *Serratia marcescens* [5], and dissemination of carbapenemase-producing Enterobacterales [6] have been reported in animal healthcare facilities, all related to poor infection prevention and control. Even so, there is a lack of evidence-based threshold values for acceptable environmental bacterial load to minimize the risk of HAI in both animal and human healthcare facilities.

Animals, animal owners and staff bring more or less pathogenic microorganisms into animal healthcare facilities. Bacteria can then be transmitted from e.g. surfaces, the air, humans, or directly between the animals. Therefore, knowledge about the presence and amount of viable and potentially pathogenic bacteria in the environment and how the bacterial load changes during various daily activities is helpful in establishing optimal hygiene routines to prevent infection transmission and HAI. People and animals together with e.g. air-conditioning, heating and ventilation systems as well as outdoor factors, including air quality are important sources of airborne microorganisms in the indoor environment [7]. Thus, it may be assumed that the indoor environment in animal healthcare facilities may vary with geographic location, and local studies are therefore needed, ideally taking also seasonal changes into account. Only a few studies, mainly outside of Europe, have reported data on bacterial loads in animal healthcare facilities [8–12]. Also, most studies have only reported bacterial load at one time point. Only one study investigated the bacterial load before and during clinical procedures, e.g. surgery [9] despite the usefulness of knowing how the bacterial load changes depending on activity and cleaning procedures.

In addition to quantification and bacterial species identification, genetic mapping can be used to trace sources of infectious disease outbreaks and for comparison between outbreaks. Knowledge of the environmental microflora composition, possible genetic relationships between bacteria (outbreak and/or house flora) and factors influencing these parameters can support tailored interventions to improve hygiene routines to lower the risk for HAI.

The aims of this study were to:

1. Quantify and identify the environmental bacterial load in air and on surfaces in one operating room and one ultrasound room in a small animal hospital in Sweden,
2. Compare the bacterial load with threshold values suggested for use in human healthcare facilities,
3. Characterise the genetic relationship between selected bacterial species to assess clonal dissemination, and
4. Investigate factors associated with bacterial load during surgery.

## Methods

### Study design

This prospective observational study was carried out in a small animal hospital in Sweden, with approximately 30,000 patient appointments per year. In February 2019, a pilot study was carried out to identify relevant sampling locations for the main study taking practical considerations into account, including that the data collector would not interfere with the workflow. The data collection for the main study took place in May 2019 to June 2020.

### Data collection

**Fig. 1:**
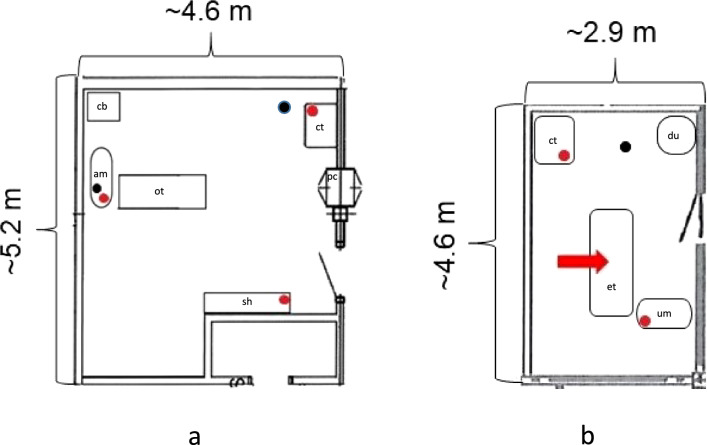
Layout of **a** the operating room (OR) and **b** the ultrasound room (UR). Red circles show location of settle plates, black circles location of negative control settle plate and dip slide, and red arrow approximate location for near-patient surface (dip slides) sampled. Distances from the midpoint of the surgical table (OR) to the location of settle plates were: ~ 1.6 m (anaesthesia machine), ~ 2 m (computer table), and ~ 2 m (shelf). Distances from the location for near-patient surface sampling (UR) to location of settle plates were: ~ 2.5 m (computer table) and ~ 1 m (ultrasound machine). Settle plate sampling locations were at a height of ~ 0.9-1.1 m. Equipment (size not according to scale): *am* anaesthesia machine, *cb* cupboard, *ct* computer table, *du* drawer unit, *et* examination table, *ot* operating table, *pc* pass-through cabinet, *sh* shelf, *um* ultrasound machine

The first author was responsible for collecting data, except in November 2019 when a trained hospital staff member collected data twice in the operating room (OR). Settle plates (14 cm in diameter; 80 mL Tryptic Soy Agar (TSA); produced in-house under aseptic conditions) were used for passive air sampling and dip slides (Envirocheck® Dip Slide DC Disinfection Control, 9.4 cm^2^ per side, Merck KGaA, Darmstadt, Germany) for surface sampling. The dip slides have a CASO (tryptic soy) agar (called CASO agar further on) on one side and on the other side a CASO agar containing neutralizers that neutralizes the disinfectants hexachlorophene, mercurial compounds, halogen compounds, chlorhexidine, aldehydes and phenolic compounds (called CASO + agar further on). Neutralizers help viable bacteria that were held in bacteriostasis after disinfection to grow on the dip slide [13]. Sampling locations are shown in Fig. 1 and described in Table 1. During sampling in the OR and ultrasound room (UR), the data collector sat by the computer table, in the upper right corner in Fig. 1a, moving every hour only to exchange the settle plates. Furthermore, before the first patient of the day was admitted the data collector sat outside the OR noting staff movements in the OR. The same procedure was repeated for the UR with the addition of ensuring no staff movements in the UR during lunchtime.

**Table 1:** Dip slide sampled surfaces in the operating and ultrasound room

Room	Category of surface	Sampling surface
OR^a^	High-touch^b^	Behind the cupboard handle
OR	High-touch	Behind the handle of the pass-through cabinet
OR	Sterile field^c^	Instrument table
OR	Sterile field	Surgical drape near surgeon
OR	Sterile field	Surgical drape near anaesthesia machine
OR	Sterile field	Handle of surgical light near anaesthesia machine
OR	Sterile field	Handle of surgical light near the door
UR^d^	High-touch	Near the roller mouse, where the wrists have contact with the surface, on the anaesthesia machine
UR	High-touch	Near the keyboard, where the wrists have contact with the surface, at the computer table
UR	Near-patient^e^	In the middle of the patient positioning on the abdominal position cushion

^a^ Operating room. ^b^ Surfaces that are frequently touched by staff and patients. ^c^ The area close to the incision covered by surgical drapes, the instrument table, and the sterile surgical light handles. ^d^ Ultrasound room ^e.^ Surface in direct contact with or near the patient

### Bacterial sampling, OR

In the study, one OR and one procedure was selected to standardize the bacteriological sampling. There were five ORs in the hospital, and the selected OR was the most frequently used OR for the chosen procedure, ovariohysterectomy (OHE). Passive air sampling was carried out during both emergency and elective OHE procedures in dogs, between 8am and 10 pm. Three settle plates were placed (Fig. 1a) before each surgery, opened during the initial 2–3 min routine team review of the procedures before surgery started and left open until the incision was sutured, then immediately closed. For surgeries exceeding one hour, plates were exchanged for new ones every hour (± 2 min).

In addition, seven selected surfaces in the OR were sampled for each surgery. A dip slide was applied with a contact time of 15 s as previously described [14] on two high-touch surfaces (surfaces that are frequently touched by healthcare workers and patients as defined by Centers for Disease Control and Prevention [15]) before the patient came into the OR, and on five sterile field surfaces sampled after completed surgery (Table 1).

As negative controls, one closed settle plate was placed on a decided spot on the anaesthesia machine and one sealed dip slide on a decided spot on the floor (Fig. 1a). The negative controls were placed just before sampling of high-touch surfaces and they were removed after sampling of the sterile field.

### Bacterial sampling, UR

The selected UR was mainly used for abdominal ultrasound examinations of dogs and cats. Sampling was carried out during midmornings when both dogs and cats were examined. Settle plates were placed in two sampling locations and were exchanged for new ones approximately every hour (± 10 min) (Fig. 1b). At lunch break (35–60 min), when the room was empty, plates were also placed for sampling. A negative control, a closed settle plate, was placed on the floor at the start of the study in the morning and collected after the lunch break.

For surface sampling, three surfaces were sampled with dip slides directly after each ultrasound examination; two high-touch surfaces and one near-patient surface (Fig. 1 and Table 1). The near-patient surface, an abdominal positioner cushion, was also sampled after routine disinfection (replaced by routine cleaning during 2020) of the cushion (Table 1). A negative control, a sealed dip slide, was placed on the floor at study start in the morning and collected after the lunch break.

### Applied threshold values for bacterial loads

There are no suggested threshold values for bacterial loads for passive air sampling or surface sampling for animal healthcare facilities, so all applied threshold values are from guidelines or recommendations for human healthcare facilities. Reference threshold values from the literature for settle plates were, when needed, transformed from colony forming units (CFU)/plate/h to a more standardized measure CFU/dm^2^/h. The plate diameter was given in all references and after calculating the plate area in dm^2^ the CFU/dm^2^/h was calculated.

Reference threshold values varied with location and use of the room. For settle plates in the OR, suggested threshold values for clean surgical procedures without increased susceptibility for infections were used [16]. For settle plates in the empty OR, threshold values expressed as suggested target and alert values were used [17]. The UR was considered a medium risk environment (such as hospital wards and outpatient clinics) and since there are no suggested threshold values specifically for URs in human healthcare facilities, suggested threshold values for medium risk environment were used [18]. For surface sampling in the OR, including high-touch surfaces, the only available suggested threshold values in human medicine are from Italy. The Italian guidelines for surgical units are expressed as expected level and acceptable level in a closed OR left empty for at least 30–60 min following cleaning and disinfection after surgery [19]. The suggested threshold value for high-touch surfaces, including near-patient surfaces in human healthcare facilities used in this study, was < 2.5 CFU/cm^2^ [20–22]. Details about the different threshold values are presented in Table 2.

**Table 2:** Applied threshold values, from human healthcare, for bacterial loads

Type of sample	Room	Threshold value	Type of threshold value according to the reference	Type of publication	Reference
Passive air sample during surgery	OR^a^	≤ 19 CFU/dm^2^/h	Suggested mean value per surgery	Swedish guidelines	16
Passive air sample during surgery	OR	≤ 39 CFU/dm^2^/h	Suggested highest value during surgery	"	16
Passive air sample in an empty OR	OR	2 CFU/dm^2^/h	Suggested target value	Prospective observational study	17
Passive air sample in an empty OR	OR	5 CFU/dm^2^/h	Suggested alert value	"	17
Passive air sample in medium risk environments (e.g. hospital wards and outpatient clinics)	UR^b^	≤ 79 CFU/dm^2^/h	Suggested threshold value	Review	18
Surface sample; high-touch and sterile field	OR	≤ 0.21 CFU/cm^2^	Suggested expected level	Italian guidelines	19
Surface sample; high-touch and sterile field	OR	≤ 0.63 CFU/cm^2^	Suggested acceptable level	"	19
Surface sample; high-touch and near-patient	UR	< 2.5 CFU/cm^2^	Suggested threshold value	Prospective observational studies	20–22

^a^ Operating room. ^b^ Ultrasound room

### Bacterial culture, count, and identification

Settle plates and dip slides were incubated in 37 ± 1 °C for 48 ± 2 h in the hospital laboratory immediately after sampling, except for samples collected during Thursdays and Fridays. The latter were refrigerated until the end of the sampling day and then transported for ~ 1–1.5 h in room temperature, before incubation at 37 ± 1 °C for 48 ± 2 h in the laboratory of the Swedish University of Agricultural Sciences. Colonies were counted manually, and numbers transformed to CFU/dm^2^/h for settle plates and CFU/cm^2^ for dip slides. For settle plates total CFU/dm^2^/h per sampling location, per surgery/midmorning with patients in the UR, was calculated using Eq. 1. Morphology was noted and colonies with different morphology originating from plates from the same surgery were subcultured on bovine blood agar, 5% (B341960; National Veterinary Institute (SVA), Uppsala, Sweden) at 37 ± 1 °C for ~ 24 h. Isolates with poor growth were incubated for another ~ 24 h. Due to excessive growth on many UR plates and therefore too many isolates to handle in the study, colonies for subculture were selected from a period of sampling, one or two midmornings, instead of every sampling day. For frequently occurring colony types (with similar morphology), multiple colonies were subcultured, while from rarely occurring colony types, one colony was selected. Bacterial species identification was performed by analysing each isolate in duplicate (technical replicates) using Matrix Assisted Laser Desorption – Time Of Flight (MALDI-TOF) (Bruker Daltonics, Billerica, MA, USA). If identification failed, formic acid (70%) was added to increase the chance of genus/species identification [23]. Colonies that did not grow after 48 h or that were unidentified by MALDI-TOF were classified as genus/species unknown.

Equation 1. Calculation of total CFU/dm^2^/h per sampling location, per surgery/midmorning in the UR

1 $$\left( {CFU_1 + CFU_2 + \cdots + CFU_n } \right) \div \left( {0,7^2 \times\pi } \right) \div \left( {\frac{{T_1 + T_2 + \cdots + T_n }}{{60}}} \right)$$

CFUn is the number of CFU on settle plate *n* and Tn the time in min settle plate *n* was kept open. The number of plates varying from 1 to 5.

### Antibiotic susceptibility testing

As an initial phenotyping method, antimicrobial susceptibility testing was performed on frequently detected staphylococci and staphylococci known to carry resistance of particular interest, such as methicillin resistance. Single colonies were inoculated on 5% bovine blood agar (B341960; SVA), incubated for ~ 24 h at 37 ± 1 °C and tested by broth microdilution (Mueller Hinton broth 321,300, SVA) according to the Clinical and Laboratory Standards Institute guidelines, using microdilutions panels (ThermoFisher, Waltham, MA, USA, Sensititre STAFSTR). As a quality control *S. aureus* CCUG 15915, (ATCC 29213) was used. The panel included the following substances; penicillin, cephalothin, cefoxitin, enrofloxacin, fusidic acid, erythromycin, clindamycin, gentamicin, nitrofurantoin, tetracycline, and trimethoprim/sulphmethoxazole. Penicillinase-production in staphylococci was tested by the cloverleaf test [24].

### Whole genome sequencing

Isolates were subcultured from single colonies to ensure pure cultures, inoculated on 5% bovine blood agar (B341960; SVA) and incubated for ~ 24 h at 37 ± 1 °C. DNA was prepared by mixing bacterial colonies with 190 mL G2 buffer (EZ1 DNA Tissue Kit; Qiagen, Hilden, Germany), adding 10 µl lysostaphin (5 mg/mL) and centrifuged at 350 rpm for 1 h 30 min at 37 °C, as slightly modified from the manufacturer’s instructions for pretreatment of gram-positive bacteria. DNA was extracted using the IndiMag Pathogen kit (Indical) on a Maelstrom-9600 automated system. Library preparation was performed using Nextera chemistry, and sequencing performed as 2 × 150 bp paired-end reads using an Illumina NovaSeq instrument at SciLifeLab Clinical Genomics, Solna, Sweden. Samples were assembled, typed with multilocus sequence typing (MLST) and screened for resistance genes. Samples with the same multilocus sequence type or allele combination were compared with single nucleotide polymorphism (SNP)-analysis to identify possibly related isolates. All *S. capitis* samples were compared with each other as no MLST scheme for the species was available. Details including program versions and parameters are provided in Additional file 1.

### Factors associated with bacterial load during surgery

Staff movements, patient data and other information connected to the surgery were registered: temperature and humidity; staff movement from incision to closed incision; number of staff; opening of the door; staff walking in or out of the OR; air change/h. Staff movements were registered as spaghetti diagrams. To translate movement into numeric values for statistical analyses movements were categorised as short, medium, or long. Short movements without actual walking were defined as 0 movement, medium long movements (~ < 2 m) as 1 movement and long movements (~ > 2 m) were defined as 2 movements. The OR had a turbulent airflow ventilation system. Air change/h was based on a mandatory ventilation control 27 May 2019 as part of the routine quality assurance procedures. The bacterial load in the OR was based on three outcomes: bacterial load on the computer table, the anaesthesia machine, and the shelf. Factors assessed for potential association with these outcomes were: acute/elective surgery, number of staff in the OR, degree of staff movement during surgery, surgery length, door openings and persons in and out of the OR.

### Hygiene routines

The hospital’s OR hygiene routines included aseptic preparation of patient and staff, compliance with correct protective wear (surgical cap and mask for everyone, sterile surgical gown, and sterile gloves for surgeon/-s). Before surgery, the sterilisation wrapping used for the instrument set, was confirmed to be intact. Special scrubs with tight-fitting cuffs were introduced by the animal hospital as an update in the IPC routine during the study period, but not worn by everyone during all surgeries.

### Data analysis

Microsoft® Excel 2016 (16.0.5134.1000) (Microsoft Corporation, Redmond, Washington, USA) was used for data management and descriptive statistics. Linear regression was performed for the association between each bacterial load outcome in the OR (measured at three sampling locations with settle plates) and potential explanatory variables in univariate analyses. Skewness and kurtosis tests for normality were used to evaluate the normality assumption using the residuals. Regression analysis was done using Stata SE 16.1 (StataCorp LLC, 4905 Lakeway Drive, College Station, Texas 77,845 USA). The Bonferroni correction was applied adjusting the P-value for statistical significance by dividing 0.05 with the number of analyses to compensate for multiple analyses and avoid overestimation of statistically significant results. The number of analyses were three outcomes * five explanatory factors = 15, thus the P-value for statistical significance was set to 0.05/15 = 0.0033.

## Results

### Number and type of procedures

**Fig. 2:**
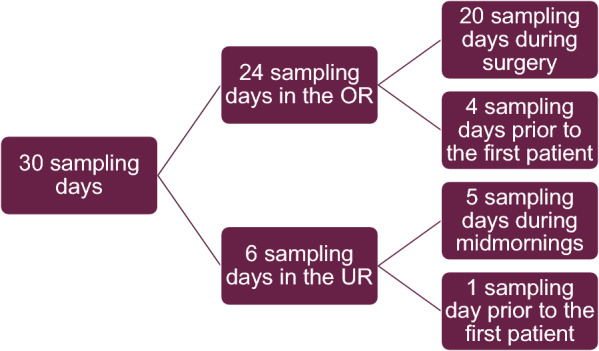
Sampling days in the study. OR = operating room, UR = ultrasound room, prior to the first patient = before the first patient of the day, during midmornings = sampling during the part of the midmorning when patients were examined

Sampling took place during 30 days, for details see Fig. 2. In the OR, data was collected on four occasions prior to the first patient of the day and during 27 OHE procedures. Twenty-one OHEs were emergency procedures, i.e. pyometra (n = 19, 1 ruptured), hydrometra (n = 1), and metritis (n = 1), the remaining were elective OHE.

**Fig. 3:**
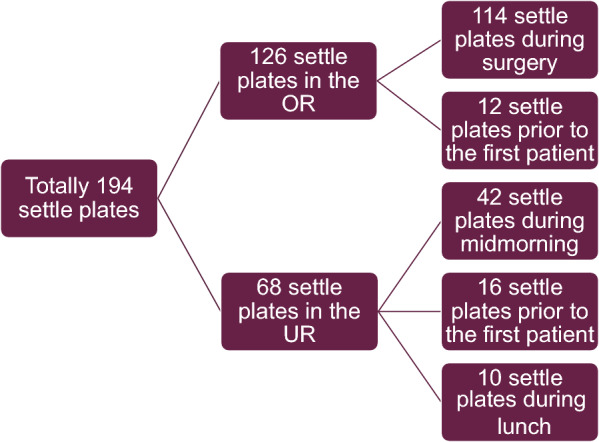
Settle plates used in the study. OR = operating room, UR = ultrasound room, prior to the first patient = before the first patient of the day, during midmornings = sampling during the part of the midmorning when patients were examined, during lunch = when the UR was empty during lunch time

**Fig. 4:**
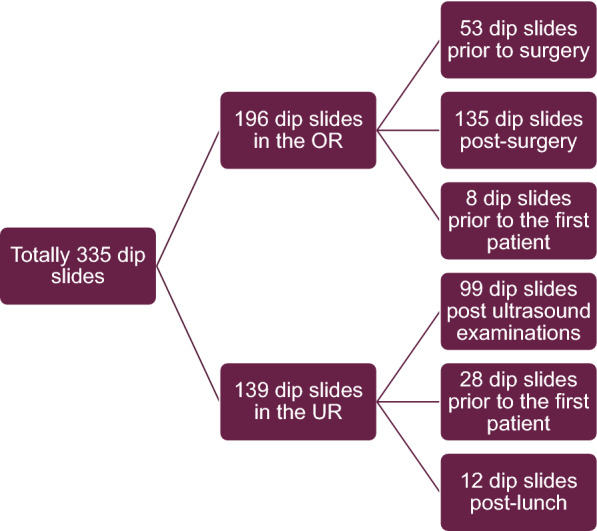
Dip slides used in the study. OR = operating room, UR = ultrasound room, prior to surgery = during preparations for the surgery, post-surgery = immediately after the surgery finished, prior to the first patient = before the first patient of the day, post ultrasound examinations = after the ultrasound examination of each patient including before and after decontamination of the abdominal position cushion, post-lunch = when the UR had been empty during lunch time and before the next patient arrived

UR data was collected during five midmornings, from the hour prior to the first patient of the day until the end of lunch break, and one morning only prior to the first patient of the day. The ultrasound patients included 12 dogs and 13 cats, most of the patients underwent an abdominal ultrasound examination while the eye was examined in one case and the neck/chest in another. Invasive sampling was performed on spleen (n = 1), liver (n = 1) and prostate (n = 1). In the study 194 settle plates and 336 dip slides were used for sampling, for details see Figs. 3 and 4.

### Bacterial load

S*taphylococcus* spp. and *Micrococcus* spp. were the dominating bacterial genera in both the OR and the UR (Tables 3 and 4, and Additional file 2).

**Table 3:** Bacterial air sampling with settle plates during surgery and ultrasound examination

Room	Sampling location	Median^a^ (25^th^-75^th^ percentile) CFU/dm^2^/h	Bacteria^b^
OR^c^	Anaesthesia machine (38^d^/29^e^)	3 (2–6)	**48.3% *Staphylococcus* spp.** (21.4% *S. epidermidis*, 14.3% *S.* spp., 14.3% *S. hominis*, 14.3% *S. pseudintermedius*, 14.3% *S. saprophyticus*, 7.1% *S. capitis*, 7.1% *S. caprae*, 7.1% *S. warneri*), **17.2% *Micrococcus* spp.** (100% *M. luteus*)
OR	Computer Table (38/61)	13 (8–18)	**42.6% *Staphylococcus* spp.** (26.9% *S.* spp., 26.9% *S. epidermidis*, 23.1% *S. capitis*, 11.5% *S. hominis*, 3.8% *S. cohnii*, 3.8% *S. equorum*, 3.8% *S. saprophyticus*), **26.2% *Micrococcus* spp.** (81.3% *M. luteus*, 12.5% *M. flavus*, 6.3% *M. cohnii*)
OR	Shelf (38/47)	9 (5–11)	**38.3% *Staphylococcus* spp.** (38.9% *S.* spp., 22.2% *S. capitis*, 16.7% *S. epidermidis*, 11.1% *S. aureus*, 5.6% *S. hominis*, 5.6% *S. lugdunensis*), **34.0% *Micrococcus* spp.** (68.8% *M. luteus*, 12.5% *M. flavus*, 6.3% *M.* spp., 6.3% *M. lylae*, 6.3% *M. terreus*)
UR^f^	Computer Table (21/53)	31 (30–38)	**35.8% Staphylococcus spp.** (36.8% *S. epidermidis*, 21.5% *S.* spp., 15.8% *S. capitis*, 15.8% *S. hominis*, 5.3% *S. equorum*, 5.3% *S. saprophyticus*), **26.4% *Micrococcus* spp.** (100% *M. luteus*)
UR	Ultrasound machine (21/55)	32 (31–38)	**36.4% Staphylococcus spp.** (30% *S. hominis*, 25% *S.* spp., 20% *S. epidermidis*, 10% *S. lugdunensis*, 5% *S. aureus*, 5% *S. capitis*, 5% *S. petrasii*), **18.2% *Micrococcus* spp.** (60% M. luteus, 20% M. spp., 20% M. flavus), **14.5% *Bacillus* spp.** (50% *B.* spp., 12.5% *B. licheniformis*, 12.5% *B. megaterium*, 12.5% *B. pumilus*, 12.5% *B. weihenstaphanensis*)

^a^ Median bacterial load per sampling occasion (surgery or midmorning in the UR). ^b^ Frequently (> 10%) occurring bacteria, includes samples taken before, during and after procedures. ^c^ Operating room. ^d^ Number of plates for bacterial count. ^e^ Number of isolates for bacterial identification. ^f^ Ultrasound room

**Table 4:** Bacterial surface sampling with dip slides during surgery and ultrasound examination

Room	Surface	Medium	Median^a^ (25^th^-75^th^ percentile) CFU/cm^2^	Bacteria^b^
OR^c^	High-touch^d^ (53^e^/23^f^)	CASO^g^	0 (0–0)	**60.9% *Staphylococcus* spp.** (35.7% *S. epidermidis*, 21.4% *S. hominis*, 14.3% *S. capitis*, 7.1% *S.* spp., 7.1% *S. haemolyticus*,7.1% *S. pseudintermedius*, 7.1% *S. warneri*)
		CASO + ^h^	0 (0–0.11)	
OR	Sterile field^h^ (135/30)	CASO	0 (0–0)	**56.7% *Staphylococcus* spp.** (35.3% *S. hominis*, 29.4% *S.* spp., 11.8% *S. epidermidis*, 11.8% *S. pseudintermedius*, 5.9% *S. haemolyticus*, 5.9% *S. saprophyticus*), **20% *Micrococcus* spp.** (100% *M. luteus*)
		CASO +	0 (0–0)	
UR	High-touch (50/34)	CASO	0.21 (0.11–0.53)	**58.8% *Staphylococcus* spp.** (45% *S. hominis*, 25% *S.* spp., 15% *S. epidermidis*, 10% *S. haemolyticus*, 5% *S. xylosus*)
		CASO +	0.53 (0.24–1.33)	
UR^i^	Near-patient^j^ after examination (25/22)	CASO	0.21 (0–0.85)	**45.5% *Staphylococcus* spp.** (30% *S. saprophyticus*, 20% *S.* spp., 10% *S. aureus*, 10% *S. capitis*, 10% *S. equorum*, 10% *S. felis*, 10% *S. warneri*), **18.2% *Macrococcus* spp.** (50% *M. canis*, 25% *M. brunensis*, 25% *M.* spp.), **13.6% *Bacillus* spp.** (66.7% *B.* spp., 33.3% *B. licheniformis*)
		CASO +	0.43 (0.11–1.91)	
UR	Near-patient after decontamination (24/12)	CASO	0 (0–0.03)	**41.7% *Bacillus* spp.** (60% *B. cereus*, *B.* spp. 20% *B. licheniformis*), **25% *Staphylococcus* spp.** (33.3% *S. capitis*, 33.3% *S. felis*, 33.3% *S. haemolyticus*), **16.7% *Kocuria* spp.** (100% *K.* spp.)
		CASO +	0.11 (0–0.27)	

^a^ Median bacterial load per dip slide. ^b^ Frequently (> 10%) occurring bacteria, includes samples taken before, during and after procedures. ^c^ Operation room. ^d^ Surfaces that are frequently touched by staff and patients. ^e^ Number of dip slides for bacterial count. ^f^ Number of isolates for bacterial identification. ^g^ CASO (tryptic soy) agar. ^h^ CASO agar containing neutralizers that neutralize hexachlorophene, mercurial compounds, halogen compounds, chlorhexidines, aldehydes and phenolic compounds. ^i^ The area close to the incision covered by surgical drapes, instrument table and sterile surgical light handles. ^j^ Ultrasound room. ^k^ Surface in direct contact with or near the patient

### Passive air sampling, settle plates

For the passive air sampling in the OR, the bacterial load varied depending on the use of the room, samples were taken before surgery, during preparation of the room, and during the surgeries. In the empty OR (1 sampling occasion) the bacterial load on all settle plates was below the suggested target value [17]; varying between 0 and 1 CFU/dm^2^/h, with a median of 0 CFU/dm^2^/h. The bacterial load when staff prepared the OR prior to the first surgery of the day (3 sampling occasions) was higher and varied between 0 and 13 CFU/dm^2^/h with a median of 2 CFU/dm^2^/h. Forty-four percent of the samples were below the suggested target value and 67% below the suggested alert value [17]. During surgery, the bacterial loads on settle plates were all below both the suggested mean value per surgery and the suggested highest value [16] (Tables 2 and 3). Additional bacterial load data is provided in Additional file 3.

In the empty UR, prior to the first patient, the bacterial load was between 0 and 1 CFU/dm^2^/h (1 sampling occasion). The bacterial load while staff were preparing for the day, was between 2 and 15 CFU/dm^2^/h (6 sampling occasions) with a median of 5 CFU/dm^2^/h. During midmornings when patients were examined, 95% of the settle plate samples were below the suggested threshold value for medium risk environments (e.g. human hospital wards and outpatient clinics) [18] (Tables 2 and 3). The two samples with bacterial load exceeding the threshold value were taken during the same data collection hour, one plate in each sampling location. During lunch (empty UR) the bacterial load was between 0 and 4 CFU/dm^2^/h (5 sampling occasions) with a median of 1 CFU/dm^2^/h. Additional bacterial load data is provided in Additional file 3.

**Fig. 5:**
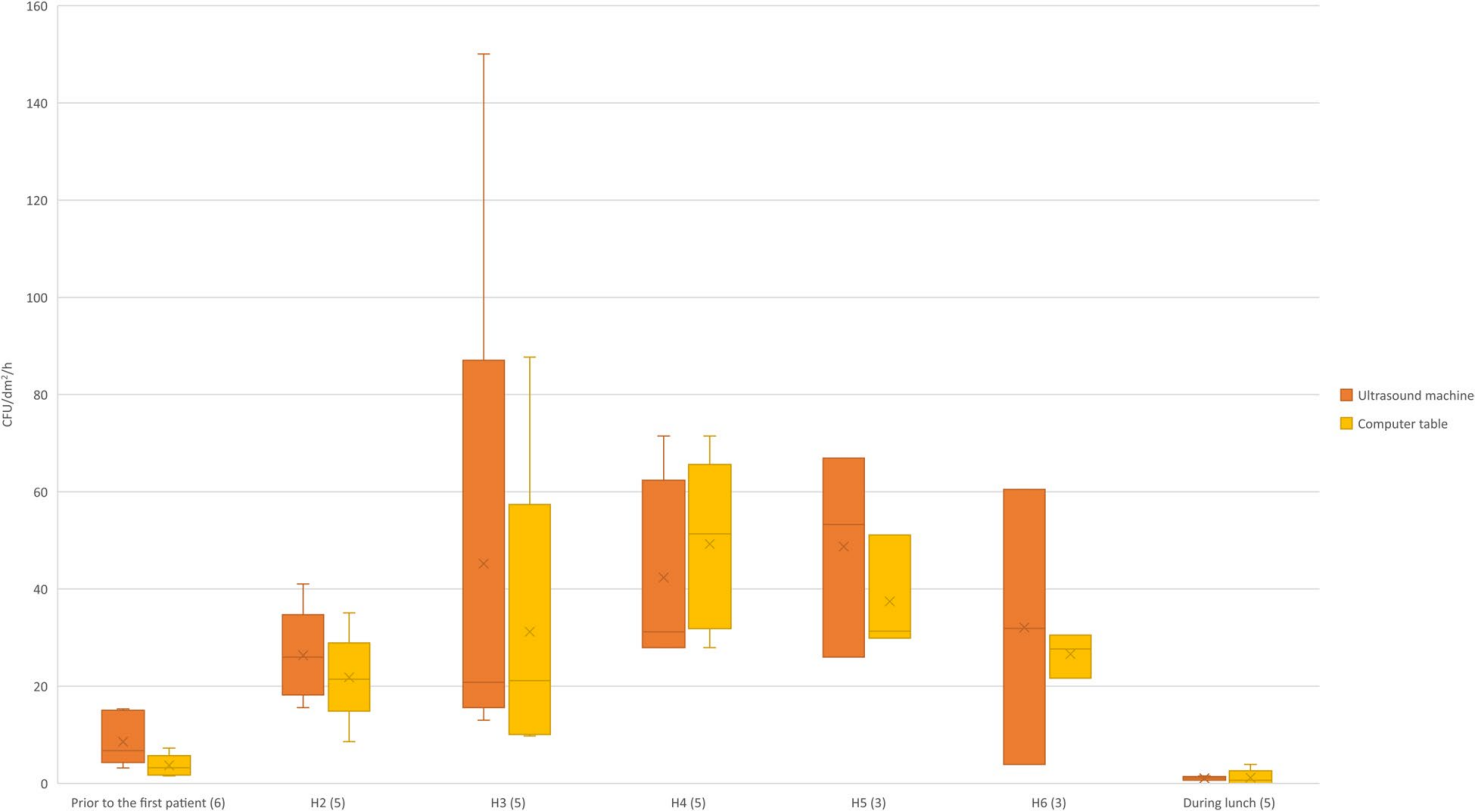
Bacterial load, in passive air samples, during mid mornings in the ultrasound room. Bacterial load reported as CFU/dm^2^/h in passive air samples from settle plates placed on the ultrasound machine and the computer table in an ultrasound room. During H2-H6 patients were present in the room. Number of settle plates on the ultrasound machine and the computer table are presented within brackets. The x marks the mean, and the line marks the median at each time slot (60 ± 10 min for all time slots but lunch break which varied between 35 and 60 min) and sampling locations

The change in bacterial load in the UR is presented in Fig. 5. In the hour before arrival of the first patient in the UR when staff were preparing for the day, the bacterial load on settle plates was low. A numerical increase in bacterial load was seen during the first hours of ultrasound examinations followed by a decrease later during the midmorning. During lunch, when the UR was empty, the bacterial load was lower than during the hour before the arrival of the first patient.

For the negative controls, some were suspected to have been contaminated during production or handling. In the OR, nine of 31 negative controls (six from samplings during surgery and three from sampling in the empty OR) were contaminated with a few colonies. In the UR, one of six negative controls was contaminated with one colony. *Sphingomonas paucimobilis* was identified as one of the contaminating bacterial species and colonies with similar morphology were therefore excluded from the bacterial counts on the same batch of plates.

### Surface sampling, dip slides

Most of the surface samples from the OR and the UR were below the suggested threshold values. In the OR, using disinfectant neutralizers (CASO + agar), 89% of high-touch samples and 93% of sterile field met the expected level (Tables 2 and 4). The acceptance level was met to 98% on high-touch surfaces and to 99% in the sterile field. In total, 14 of 16 of the high-touch surface samples taken prior to the first patient of the day were negative and all samples met the expected level. Additional bacterial load data is provided in Additional file 4.

In the UR, using disinfectant neutralizers, 88% of high-touch surface samples and 100% of the near-patient surfaces after decontamination met the threshold value (Tables 2 and 4). All surface samples taken prior to the first patient of the day or at the end of the lunch break met the threshold value. Additional bacterial load data is provided in Additional file 4.

### Antibacterial resistance and sequencing

A total of 78 isolates of frequently occurring species were frozen (−70/−80 °C) for further analyses. Of them, 51 frequently detected staphylococci (*S. aureus, S. capitis, S. epidermidis, S. hominis, and S. pseudintermedius*) and staphylococci known to carry resistance of particular interest, such as methicillin resistance (e.g. *Staphylococcus aureus*, cefoxitin MIC > 4 mg/L and *S. pseudintermedius*, oxacillin MIC > 0.5 mg/L) were selected for antimicrobial susceptibility testing. No suspected methicillin-resistant (MR) isolates were detected in the 51 analysed staphylococci isolates. Thirty-six of the 51 isolates were allocated to ten phenotypes based on the antibiotic susceptibility pattern and selected for sequencing. Of the 36 staphylococci isolates, 35 were successfully analysed by whole genome sequencing. There was no indication of clonal relationship among these isolates. Multilocus sequence types and allelic profiles are presented in Additional file 5. Identification of resistance genes was not the main purpose of the study. However, the sequencing revealed genes conveying resistance to disinfectants, e.g. quaternary ammonium compounds (QACs) and chlorhexidine in 12 of 35 isolates: *qacA* was found in *S. epidermidis* (n = 1) and *S. hominis* (n = 4), *qacB* was found in *S. capitis* (n = 3) and *S. hominis* (n = 3), and *qacJ* was found in *S. epidermidis* (n = 1). A majority of the isolates with genes conveying resistance to biocides were collected in the OR (10/24) and only a few in the UR (2/11). Genes conveying resistance identified by sequencing are presented in Additional file 6.

### Factors potentially related to the bacterial load in the OR

Table 5 shows median, min, max, 25^th^ and 75^th^ percentile of surgery time, opening of door, staff walking in or out of the door, staff movement, number of staff, temperature, and relative humidity in OR. During most (74%) surgeries, an increase in temperature and a decrease in humidity were observed. The air change was ~ 21 changes/h which meets the suggested ventilation rate of 17–20 changes/h to decrease the microbial air contamination [25].

**Table 5:** Observations and data during surgery

	Median (25^th^-75^th^ percentile)	Min	Max
Surgery time (min)	49 (37.5–68)	16	135
Opening of door (n^a^)	5 (2–9)	0	25
Staff walking in or out (n)	3.5 (2–7)	0	23
Staff movement (n)	57 (40.8–92)	4	139
Staff (n)	4 (3–4.5)	3	6
Temperature^b^ (°C)	23.0 (22.0–25.6)	21.4	28.1
Relative humidity^c^ (%)	32.5 (24.4–41.7)	13.3	58.4

^a^ Number of occasions. ^b^ Median temperature based on lowest and highest temperature for each surgery. ^c^ Median humidity based on lowest and highest humidity for each surgery

**Fig. 6:**
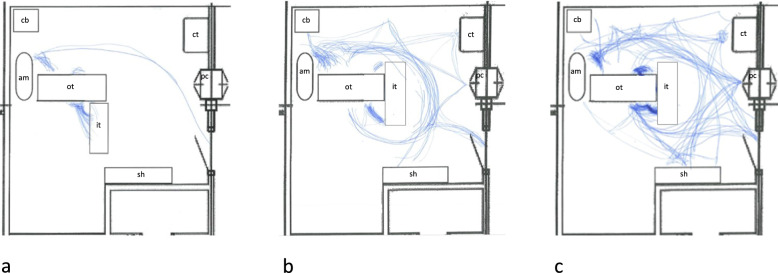
Spaghetti diagrams of movements in the operating room. Spaghetti diagrams showing examples of **a** low-grade movement **b** moderate grade movement, and **c** high-grade movement during surgery. Equipment (size not according to scale): *am* anaesthesia machine, *cb* cupboard, *ct* computer table, *it* instrument table, *ot* operating table, *pc* pass-through cabinet, *sh* shelf

The number of movements in the OR, described in spaghetti diagrams (Fig. 6), varied considerably between the surgeries, from only four movements to 139 movements. Correlation analysis showed a strong correlation between the number of times the door was opened and the number of persons going in and out of the room, therefore only one of these factors was evaluated as potentially associated with the outcome (door opening). None of the explanatory factors were significantly associated with the outcomes at the set P-value of 0.0033.

## Discussion

This study shows that it is possible for small animal hospital ORs to achieve bacterial loads below the threshold values suggested for use in human healthcare facilities thus posing a reduced risk for post-operative infections. The low bacterial load prior to and between surgeries implies that the hospital’s hygiene routines were sufficient and reduced the risk of environmental transmission of bacteria between patients. Similar conclusions can be drawn for the UR where 84–100% of the samples were below the threshold values suggested for use in human healthcare facilities.

In this study, bacterial loads prior to surgery were considerably lower than the values reported in a study of veterinary operating rooms [8], where 83% of the active air samples were above the suggested alert value for active air samples in human healthcare facilities [17]. Active air sampling is assumed to capture more bacteria, compared to passive air sampling. Results from passive and active air sampling have been shown to correlate in ORs with a turbulent mixed airflow, both in empty ORs and during surgery [26]. In a recent study, including ORs with different ventilation systems (~ 75% with unidirectional airflow), a correlation between passive and active air sampling was also shown [27]. The study also showed that the EU GGMP relationship between passive and active air 1:8 could be considered valid for operating rooms [27]. For the suggested target and alert values, the relationship is 1:12 respectively 1:11 indicating the suggested target values for active air sampling could be a bit easier to meet [17]. Based on this the difference between the result in the present study and the other study investigating the bacterial load prior to surgery [8] can be assumed to be accurate. Similar results as those presented in that study [8] were presented in another study [9]. In that study [9] the reported geometrical mean bacterial loads per operation room was however below the suggested threshold value for clean surgical procedures without increased susceptibility for infections [16], although type of surgery was not described. The number of surgeries included for sampling were fewer than in our study and they only sampled one time for 10 min per surgery, thus comparison is difficult [9]. According to the Swedish guidelines [16] it is recommended to do repeated active air samplings during surgery, where 3 to 4 samplings is preferable. The higher bacterial load found during the procedures in our study is in line with that human and animal presence may be one of the greatest sources of the airborne microbial load in the indoor environment [7].

Compliance with the hygiene routines during clinical procedures is likely one of the most important factors for limiting transmission of infectious agents. Hygiene routines include among other things adequate environmental cleaning and, when needed, disinfection between patients as well as pre-operative skin cleaning and disinfection. The importance of adequate hygiene routines in veterinary OR was shown in a study identifying chlorhexidine solution, in which gauze was pre-impregnated, used for pre-operative skin disinfection as the source of a serratia-outbreak of HAI [5]. The compliance with correct wearing of cap and mask was high (> 90%, data not shown) in the studied hospital compared to the results in a study in human healthcare facilities where the proportion of correctly worn face masks were only 65% [28].

It is likely that adequate air change and the observed high compliance with the small animal hospital’s OR hygiene routines (data not shown) are contributing reasons for the overall low bacterial load reported in our study. As expected, the bacterial load on settle plates and dip slides in the UR was considerably higher compared to the OR, as UR hygiene was adapted to non-invasive procedures. Nevertheless, the risk of spreading bacteria in a diagnostic imaging department should not be neglected. To reduce the transmission risk, ultrasound examinations of suspected or known infectious patients could be performed in separate rooms, or as the last patient of the day in the regular UR and followed by sanitation.

The bacterial flora was dominated by *Staphylococcus* spp*.* and *Micrococcus* spp*.,* especially in the OR and on high-touch surfaces. *Bacillus* spp. was also frequently detected. These findings are comparable to results from other studies performed in animal healthcare facilities [9–11]. *Micrococcus luteus, S. capitis, S. epidermidis, S. haemolyticus, S. hominis, S. warneri,* and *Kocuria* spp*.* are all common bacteria in the human skin microbiome [29, 30]. Findings of such bacteria might pose a lesser risk to patients. *Kocuria* spp., *Macrococcus* spp*.*, *Micrococcus* spp., and *Staphylococcus* spp. including *S. pseudintermedius* are common in the canine skin flora [30–33]. Similarly, *Micrococcus* spp., and *Staphylococcus* spp. including *S. pseudintermedius* are common in the feline skin flora [34, 35]. *Bacillus* spp. has been reported to contaminate canine fur [32]. Hence, some of the most commonly detected *Staphylococcus* spp. in this study probably originated from staff (OR and UR) and/or animal owners (UR), while *S. pseudintermedius, Macrococcus* spp*.* more likely originated from the patients*. Kocuria* spp. and *Micrococcus* spp. could be of either human or animal origin. *Bacillus* spp. likely originated from the hospital environment.

There was no indication of clonal spread of the sequenced *Staphylococcus* spp., indicating that hygiene routines may have had the desired effect, i.e. old bacteria vanished with cleaning and new ones were introduced by staff, animal owners, and patients. Resistance to chlorhexidine has previously been reported in bacteria found in animal healthcare facilities [5]. The finding of genes conveying resistance to chlorhexidine as well as QACs in the present study is interesting since such disinfectants are often used in animal healthcare facilities. If chlorhexidine and/or QACs are used in the clinic, pathogenic or opportunistic bacteria with such resistance traits may persist in the environment entailing an increased risk of HAI [36]. However, a newly published systematic review and meta-analysis showed there is no evidence of reduced susceptibility to chlorhexidine in staphylococci or streptococci of human origin [37]. In vitro studies have demonstrated multiple mechanisms for the development of resistance to QACs [38] although resistance in human clinical settings seems uncommon. Our finding of genes coding for resistance to QACs indicates that use of disinfectants may select for resistant bacteria in the veterinary clinical environment.

A limitation of the study was that the settle plates were produced under aseptic, but not sterile, conditions and several negative controls were found to be contaminated. Thus, the reported bacterial load might be slightly higher than the actual bacterial load, but as the negative controls had only a few contaminating colonies it can be assumed that this had limited impact on the overall results.

There is a need for evidence-based threshold values for animal healthcare facilities, but due to a lack of such, the present study used threshold values suggested for human healthcare facilities. In our study, most of the bacterial loads were below these values which can be assumed to reduce the risk for HAI. However, the threshold level to prevent HAI is still unknown. Future studies may investigate threshold levels for animal healthcare facilities, to ensure relevant and safe bacterial loads for the patients.

## Conclusions

This study presents a generally low bacterial load in both the OR and UR, indicating a low risk of transmission of bacteria from the clinical environment. The results show that it is possible to achieve bacterial loads in the OR in small animal hospitals below the threshold values suggested for use in human healthcare facilities and thus posing a reduced risk of HAI. Bacteria carrying genes conveying resistance to disinfectants indicate that resistant bacteria can persist in the clinical environment, with increased risk for HAI.

### Supplementary Information

Additional file 1. Program versions and parameters for bioinformatic analysis. Sample reads were trimmed with Trimmomatic [1] and checked for contamination with Kraken2 [2]. For whole genome assembly the reads were normalized with the BBNorm tool from the BBTools suite [3] and assembled with Unicycler [4]. The assembly was then used for multi-locus sequence typing (MLST) using PubMLST schemes [5–9] and resistance gene identification with ResFinder [10–12]. Samples with the same sequence type or allele combination were compared with single nucleotide polymorphism (SNP)-analysis. SNP-analysis was performed for all S. capitis samples since there is no MLST scheme available for this species. For the SNP-analysis reads were downsampled using the reformat tool from BBTools [3] and mapped to reference genomes (Accession nrs. GCA_020740065.1, GCF_006094375.1, GCF_003812505.1, GCF_016126715.1) with Bowtie2 [13] and SAMTools [14]. SNPs were called and filtered with BCFTools [14] and an in-house python script [15].

Additional file 2. Bacterial flora identified on settle plates and dip slides in the operating room and the ultrasound room prior to procedures (including prior to the first procedure of the day), during procedures and after procedures. OR Operating room UR Ultrasound room.

Additional file 3. Additional data from bacterial air sampling with settle plates in the operating room and the ultrasound room. Description of data: CI confidence interval OR operating room UR ultrasound room.

Additional file 4. Additional data from bacterial surface sampling with dip slides in the operating room and the ultrasound room. a. confidence interval b. operation room c. surfaces that are frequently touched by healthcare workers and patients d. CASO (tryptic soy) agar e. CASO agar containing neutralizers that neutralize hexachlorophene, mercurial compounds, halogen compounds, chlorhexidines, aldehydes and phenolic compounds f. the area close to the incision covered by surgical drapes, instrument table and sterile surgical light handles g. ultrasound room h. surface in direct contact with or near the patient.

Additional file 5. Identified multilocus sequence types and allelic profiles. Description of data: OR Operating room UR Ultrasound room SP Settle plate DS Dip slide ST Sequence type. ~ n Denotes novel allele similar to a known allele n.

Additional file 6. Resistance genes identified by sequencing. Description of data: OR Operating room UR Ultrasound room SP Settle plate DS Dip slide blaZ Beta-lactam resistance qacA Disinfectant resistance qacB Disinfectant resistance qacJ Disinfectant resistance fosB Fosfomycin resistance vga (A) Streptogramin B resistance vga (A) V Streptogramin B resistance (Vga-A variant) fusB Fusidic acid resistance msr (A) Macrolide, Lincosamide and Streptogramin B resistance mph (C) Macrolide resistance.

## Data Availability

All raw sequencing data have been submitted to the European Nucleotide Archive and are available under accession number PRJEB52615. The datasets supporting the conclusions of this article are included within the article and its additional files.
