# Supplementary material for: Environmental bacterial load during surgical and ultrasound procedures in a Swedish small animal hospital
Source: Acta Vet Scand. 2024 Sep 2;66:43. doi: 10.1186/s13028-024-00768-4 (PMC11367971; doi:10.1186/s13028-024-00768-4)
Supplement: Supplementary file 1 — Additional file 1. Program versions and parameters for bioinformatic analysis. Sample reads were trimmed with Trimmomatic [1] and checked for contamination with Kraken2 [2]. For whole genome assembly the reads were normalized with the BBNorm tool from the BBTools suite [3] and assembled with Unicycler [4]. The assembly was then used for multi-locus sequence typing (MLST) using PubMLST schemes [5–9] and resistance gene identification with ResFinder [10–12]. Samples with the same sequence type or allele combination were compared with single nucleotide polymorphism (SNP)-analysis. SNP-analysis was performed for all S. capitis samples since there is no MLST scheme available for this species. For the SNP-analysis reads were downsampled using the reformat tool from BBTools [3] and mapped to reference genomes (Accession nrs. GCA_020740065.1, GCF_006094375.1, GCF_003812505.1, GCF_016126715.1) with Bowtie2 [13] and SAMTools [14]. SNPs were called and filtered with BCFTools [14] and an in-house python script [15]. [file 13028_2024_768_MOESM1_ESM.pdf]

Additional file 1: Program versions and parameters for bioinformatic analysis

Sample reads were trimmed with Trimmomatic [1] and checked for contamination with Kraken2 [2]. For whole genome assembly the reads were normalized with the BBNorm tool from the BBTools suite [3] and assembled with Unicycler [4]. The assembly was then used for multi-locus sequence typing (MLST) using PubMLST schemes [5-9] and resistance gene identification with ResFinder [10-12]. Samples with the same sequence type or allele combination were compared with single nucleotide polymorphism (SNP)-analysis. SNP-analysis was performed for all *S. capitis* samples since there is no MLST scheme available for this species. For the SNP-analysis reads were downsampled using the reformat tool from BBTools [3] and mapped to reference genomes (Accession nrs. GCA_020740065.1, GCF_006094375.1, GCF_003812505.1, GCF_016126715.1) with Bowtie2 [13] and SAMTools [14]. SNPs were called and filtered with BCFTools [14] and an in-house python script [15].

Program	Use	Version	Parameters	References
FastQC	Quality check of raw, trimmed, normalized and downsampled reads	0.11.9	NA	16
Trimmomatic PE	Read trimming	0.39	ILLUMINACLIP:NexteraPE-PE.fa:2:30:10:2:true SLIDINGWINDOW:4:20 MINLEN:36	1
BBTools - BBNorm	Read normalising for assembly	38.79	target=100	3
Kraken2	Contamination check	2.1.2 Database: MiniKraken v2 8GB 2019-04-23	--paired	2
Unicycler	Whole genome assembly	0.4.8	--mode normal	4
mlst	Multi-locus sequence typing	2.19.0 Database downloaded 2022-02-14	--minid 95 --mincov 10 --minscore 50	17

ResFinder	Identification of resistance genes	Software: 4.1.10<br>Database: 2022-02-04	ID threshold: 90 %<br>Min. length: 60 %<br>Species database "Staphylococcus aureus" for *S. aureus* samples, "Other" for other samples.	10-12
BBTools - reformat	Downsampling of reads for SNP analysis	38.79	sbt=250000000 for *S. capitis* and *S. epidermis*<br>sbt=230000000 for *S. hominis*<br>sbt=270000000 for *S. pseudointermedius*<br>sbt=290000000 for *S. aureus*	3
Bowtie2	Mapping of reads to reference sequence	2.4.4	-X 1000	13
SAMTools	BCF file conversion and sorting	1.14	NA	14
BCFTools	Mpileup generation	1.14	-O u -d 1000	14
	SNP calling	1.14	-m -v -O v --ploidy 1	
	SNP filtering	1.14	-O v -i 'QUAL > 30 && DP > N_SAMPLES*10 && INDEL = 0 && COUNT(GT[*] = ".") = 0 && COUNT(GT[*] = "1") < N_SAMPLES'	
Python script	SNP filtering based on phred-scaled genotype likelihood (PL) values	3.7.6	max(PL) – min(PL) > 100	15

References

1. Bolger AM, Lohse M, Usadel B. Trimmomatic: a flexible trimmer for Illumina sequence data. . Bioinformatics. 2014; 30:2114-20.
2. Wood DE, Lu J, Langmead B. Improved metagenomic analysis with Kraken 2. Genome Biology. 2019; 10.1186/s13059-019-1891-0.

3. Bushnell B, editor BBMap: A Fast, Accurate, Splice-Aware Aligner2014 2014-03-17; United States. Research Org.: Lawrence Berkeley National Lab. (LBNL), Berkeley, CA (United States)Sponsor Org.: USDOE Office of Science (SC).
4. Wick RR, Judd LM, Gorrie CL, Holt KE. Unicycler: Resolving bacterial genome assemblies from short and long sequencing reads. PLoS Comput Biol. 2017; 10.1371/journal.pcbi.1005595.
5. Jolley KA, Bray JE, Maiden MCJ. Open-access bacterial population genomics: BIGSdb software, the PubMLST.org website and their applications. Wellcome Open Res. 2018; 10.12688/wellcomeopenres.14826.1.
6. Solyman SM, Black CC, Duim B, Perreten V, van Duijkeren E, Wagenaar JA, et al. Multilocus sequence typing for characterization of Staphylococcus pseudintermedius. J Clin Microbiol. 2013; 10.1128/jcm.02421-12.
7. Zhang L, Thomas JC, Miragaia M, Bouchami O, Chaves F, d'Azevedo PA, et al. Multilocus sequence typing and further genetic characterization of the enigmatic pathogen, Staphylococcus hominis. PLoS One. 2013; 10.1371/journal.pone.0066496.
8. Thomas JC, Vargas MR, Miragaia M, Peacock SJ, Archer GL, Enright MC. Improved multilocus sequence typing scheme for Staphylococcus epidermidis. J Clin Microbiol. 2007; 10.1128/jcm.01934-06.
9. Enright MC, Day NP, Davies CE, Peacock SJ, Spratt BG. Multilocus sequence typing for characterization of methicillin-resistant and methicillin-susceptible clones of Staphylococcus aureus. J Clin Microbiol. 2000; 10.1128/jcm.38.3.1008-1015.2000.
10. Zankari E, Allesøe R, Joensen KG, Cavaco LM, Lund O, Aarestrup FM. PointFinder: a novel web tool for WGS-based detection of antimicrobial resistance associated with chromosomal point mutations in bacterial pathogens. Journal of Antimicrobial Chemotherapy. 2017; 10.1093/jac/dkx217.
11. Camacho C, Coulouris G, Avagyan V, Ma N, Papadopoulos J, Bealer K, et al. BLAST+: architecture and applications. BMC Bioinformatics. 2009; 10.1186/1471-2105-10-421.
12. Bortolaia V, Kaas RS, Ruppe E, Roberts MC, Schwarz S, Cattoir V, et al. ResFinder 4.0 for predictions of phenotypes from genotypes. Journal of Antimicrobial Chemotherapy. 2020; 10.1093/jac/dkaa345.
13. Langmead B, Salzberg SL. Fast gapped-read alignment with Bowtie 2. Nature Methods. 2012; 10.1038/nmeth.1923.
14. Danecek P, Bonfield JK, Liddle J, Marshall J, Ohan V, Pollard MO, et al. Twelve years of SAMtools and BCFtools. Gigascience. 2021; 10.1093/gigascience/giab008.
15. Foundation PS. Python Language Reference, version 3.7.
16. Andrews S. FastQC: A Quality Control Tool for High Throughput Sequence Data. 2012.
17. Seeman T. mlst. 2015. https://github.com/tseemann/mlst. Accessed 14 Feb 2022.
